# Gene Regulation by H-NS as a Function of Growth Conditions Depends on Chromosomal Position in *Escherichia coli*

**DOI:** 10.1534/g3.114.016139

**Published:** 2015-02-19

**Authors:** Elisa Brambilla, Bianca Sclavi

**Affiliations:** * LBPA, UMR 8113 du CNRS, Ecole Normale Supérieure de Cachan, Cachan, France; † School of Engineering and Science, Jacobs University Bremen, Bremen, Germany

**Keywords:** *E. coli*, gene position, genome organization, cellular adaptation, H-NS

## Abstract

Cellular adaptation to changing environmental conditions requires the coordinated regulation of expression of large sets of genes by global regulatory factors such as nucleoid associated proteins. Although in eukaryotic cells genomic position is known to play an important role in regulation of gene expression, it remains to be established whether in bacterial cells there is an influence of chromosomal position on the efficiency of these global regulators. Here we show for the first time that genome position can affect transcription activity of a promoter regulated by the histone-like nucleoid-structuring protein (H-NS), a global regulator of bacterial transcription and genome organization. We have used as a local reporter of H-NS activity the level of expression of a fluorescent reporter protein under control of an H-NS−regulated promoter (P*hns*) at different sites along the genome. Our results show that the activity of the P*hns* promoter depends on whether it is placed within the AT-rich regions of the genome that are known to be bound preferentially by H-NS. This modulation of gene expression moreover depends on the growth phase and the growth rate of the cells, reflecting the changes taking place in the relative abundance of different nucleoid proteins and the inherent heterogeneous organization of the nucleoid. Genomic position can thus play a significant role in the adaptation of the cells to environmental changes, providing a fitness advantage that can explain the selection of a gene’s position during evolution.

The effect of genomic position on the regulation of gene expression is a long-standing question that has been addressed in both eukaryotic and prokaryotic cells. Although for the former there are clear effects due, for example, to epigenetic chromatin organization into compact heterochromatin and more accessible and transcriptionally active euchromatin (Voss and Hager 2014), for bacterial cells the cause for a positional effect has been more elusive. Since the pioneering work of Chandler and Pritchard in 1975 (Chandler and Pritchard 1975) it has been clear that there is a difference in gene expression due to the copy number depending on the proximity to the origin of replication resulting from the presence of overlapping DNA replication rounds in bacterial cells. This has been proposed to be the main reason for the conservation of gene position (Couturier and Rocha 2006; Sobetzko *et al.* 2012). However, no differences subsist once the level of gene expression is normalized by the gene copy number (Schmid and Roth 1987; Sousa *et al.* 1997; Thompson and Gasson 2001; Dryselius *et al.* 2008; Block *et al.* 2012; Ying *et al.* 2014). In a recent study, however, Bryant *et al.* (2015) observed for the first time genome position−dependent effects on the *lac* promoter that can be attributed to several different factors, including the local changes in negative supercoiling due to the transcription activity of the neighboring genes and the presence of transcriptionally silent extended protein occupancy domains (tsEPODs) (Vora *et al.* 2009). Most of these studies were performed on the activity of gene expression from promoters that are regulated by specific transcription factors. Here we have asked whether the same applies for a promoter whose activity is controlled by global regulators, such as the nucleoid proteins factor for inversion stimulation (FIS) and histone-like nucleoid-structuring protein (H-NS). In addition, most of the aforementioned studies have chosen a specific growth condition for the study of gene expression in mid-exponential phase. Here we have observed how a gene’s position may affect the change of expression as the cells adapt to different growth temperatures, growth rates, and to the entry into stationary phase.

Cellular adaptation to changing environmental conditions requires the coordinated regulation of expression of large sets of genes. This regulation can take place via the activity of specific transcription factors and/or through the effects of global regulators. The latter include small metabolites, such as cAMP, ppGpp, or c-di-GMP; specific sigma factors; the set of abundant nucleoid proteins (NAPs); and changes in DNA topology due to the combined activity of transcription, DNA replication, topoisomerases, and NAP binding (Blot *et al.* 2006; Bradley *et al.* 2007; Geertz *et al.* 2011). Recent high-throughput studies have identified the genes whose expression is affected by these different regulatory factors and the binding sites of nucleoid proteins along the genome (Blot *et al.* 2006; Bradley *et al.* 2007; Peter *et al.* 2004; Lucchini *et al.* 2006; Oshima *et al.* 2006; Grainger *et al.* 2006; Wade *et al.* 2007; Cho *et al.* 2008; Kahramanoglou *et al.* 2011). Bioinformatic analysis of these results has revealed the presence of clusters of coregulated genes along the genome (Vora *et al.* 2009; Scolari *et al.* 2011; Zarei *et al.* 2013), suggesting that the level of expression of a given gene also may depend on its local environment and thus its position in the genome. The idea that different regions of the bacterial chromosome may be preferential targets for a specific subset of regulatory genes is also supported by the high level of conservation of a gene’s position with respect to the distance from the origin of replication in the family of gammaproteobacteria (Sobetzko *et al.* 2012).

To determine the extent to which chromosomal position can influence the regulation of expression of a given gene, one can place the same reporter construct at different sites along the genome. To address whether the regulation of gene expression by a global regulator such as H-NS is dependent on genomic position, we have inserted a construct consisting of an H-NS−dependent promoter (P*hns*) regulating yellow fluorescent protein (YFP) expression at six different mirror sites across the two replichores. The use of this promoter not only allows us to probe the local level of H-NS activity but also allows us to obtain some information on how the *hns* gene itself might be regulated and the effect it would have if moved away from its evolutionary conserved position in the genome, near the terminus of replication.

H-NS is a well-characterized, highly abundant (~20,000 copies), nucleoid organizing protein that can affect the expression of hundreds of genes (Dorman 2007). Notably, gene regulation by H-NS plays an important role in the response to stress, such as acid or cold shock (La Teana *et al.* 1991; Giangrossi *et al.* 2005). H-NS has a high affinity for AT-rich regions (Tanaka *et al.* 1991) and can also recognize a specific consensus sequence (Lang *et al.* 2007). The high-affinity sites can act as nucleation sites for further oligomerization. This oligomerized state can repress transcription either by trapping RNAP already bound on the DNA or inhibiting RNA polymerase binding to a promoter sequence (Schröder and Wagner 2000; Hommais *et al.* 2001; Dame *et al.* 2006; Maurer *et al.* 2009); thus, H-NS has been shown to also play a role in preventing transcription of spurious RNA from -10 sequences found within AT-rich regions (Singh *et al.* 2014). Moreover, the ability of the H-NS protein to bridge different DNA regions together contributes to compaction of the nucleoid (Dillon and Dorman 2010).

## Materials and Methods

### Chromosomal insertions

The *hns* promoter (+12 to −540 bp from the start site of transcription) was cloned upstream the yfp gene and the gene coding for resistance to chloramphenicol. Upstream of the promoter and downstream of the yfp gene T1 terminators from the *Escherichia coli rrnB* coding sequence were added to stop transcription from RNA polymerases coming from neighboring genes. The construct was amplified by polymerase chain reaction and inserted in the *E. coli* CSH50 strain in six different chromosomal positions (Table 1) with RedE/T recombination (genebridges) as described previously (Berger *et al.* 2010). The insertions were made between two convergent genes to avoid perturbations due to promoter regions of neighboring genes.

**Table 1:** Genomic position of the insertion sites used in this work

Insertion	Orientation of P*hns*-yfp	Gene1	Insertion Position	Gene2
LO: left origin	+1	*yhcN*	3,383,900	*yhcO*
RO: right origin	−1	*ytfL*	4,437,900	*ytfK*
LM: left medium	−1	*yfiF*	2,715,500	*Ung*
RM: right medium	+1	*gsK*	500,700	*ybaL*
LT: left terminus	−1	*yehA*	2,185,400	*yohN*
RT: right terminus	+1	*yccU*	1,027,600	*yccV*
LT 2	+1	*yeeJ*	2,050,100	*yeeL_1*
RT1	+1	*ycdT*	1,093,500	*insEF-4*
LT1	+1	*yegS*	2,167,700	*gatR_1*

Gene 1 and gene2 correspond to the convergent genes upstream and downstream of the reporter construct. YFP, yellow fluorescent protein.

### Plate reader assay

Cultures were grown overnight in Luria-Bertani growth medium (LB) supplemented with chloramphenicol (20 µg/mL), at 37° in a shaking incubator. Using the automated pipetting workstation Biomek 3000 (Beckman Coulter), we diluted samples 1:10000 into a 96-well plate, which were grown in triplicates at 37° or 30° inside the plate reader Victor3 (Perkin Elmer), with shaking. Samples were covered with mineral oil (Sigma-Aldrich) to avoid evaporation. The optical density of a sample measured at a wavelength of 600 nm (OD_600_) and fluorescence measurements (excitation filter = F485/14, emission filter = F535/40) were taken for each well every 5 min. The growth media used were M9 minimal media supplemented with glucose 0.4%, casaamino acids 0.5%, casaamino acids 0.2%, glucose 0.4%, and casaamino acids 0.5%.

The data obtained from the plate reader measurements were analyzed with a custom Matlab (MathWorks) program. The value of the OD_600_ for each well was normalized by subtracting the value of the well containing only the growth medium. The fluorescence measurements were normalized by subtracting the fluorescence of the wild type strain that does not contain the fluorescent reporter gene. Fluorescent protein concentration was calculated as YFP/OD_600_, and the growth rate α as $\alpha =\text{d}\left({\text{OD}}_{\text{600}}\right)/\text{dt}/{\text{OD}}_{600}$. The doubling time is then calculated as τ=ln(2)/α.

### Estimation of gene copy number

The gene copy number at a given chromosomal position can be estimated from the length of the C and D periods of the DNA replication cycle using the following equation:

$$\text{gcn}=2^{\frac{C(1-m^{\prime })+D}{\tau }}$$

where C and D are the time necessary to copy the genome and to complete cell division from the time of replication termination respectively, m’ is the distance from the origin (1 for terminus, 0 for the origin) and τ is the doubling time. The C and D periods used where the ones determined by Stokke *et al.* (2012) for a strain and growth conditions very similar to the ones used here.

### Flow cytometer

Cultures were grown overnight in LB supplemented with chloramphenicol (20 µg/mL), at 37° in a shaking incubator, and diluted in the morning 1:250 in M9 minimal media supplemented with the desired nutrients. The strains were grown in flasks at 37°, shaking. At mid-exponential phase (OD_600_ ~0.2) 2-mL samples were harvested, washed with filtered phosphate-buffered saline, and fixed with 4% formaldehyde (Sigma-Aldrich) at room temperature for 15 min, washed again with phosphate-buffered saline, and then analyzed with a flow cytometer (BD FACS Calibur; BD Biosciences) using the software BD CellQuest Pro.

The voltage for forward scatter (FSC) and side scatter (SSC) were chosen so that the bacterial population was entirely on scale on an FSC *vs.* SSC plot. A nonfluorescent bacterial sample was used to appropriately set the FL1 voltage. Individual FSC, SSC and FL1 histograms were checked to insure that the bell-shaped populations are not cut off on the display. An event rate of ~1000 events per second was maintained to minimize the chance of coincidence and to improve population resolution. In the FSC *vs.* SSC plot, a live gate R1 was set around the bacterial population and a total of ~20,000 events inside the gate were measured.

## Results

**Figure 1:**
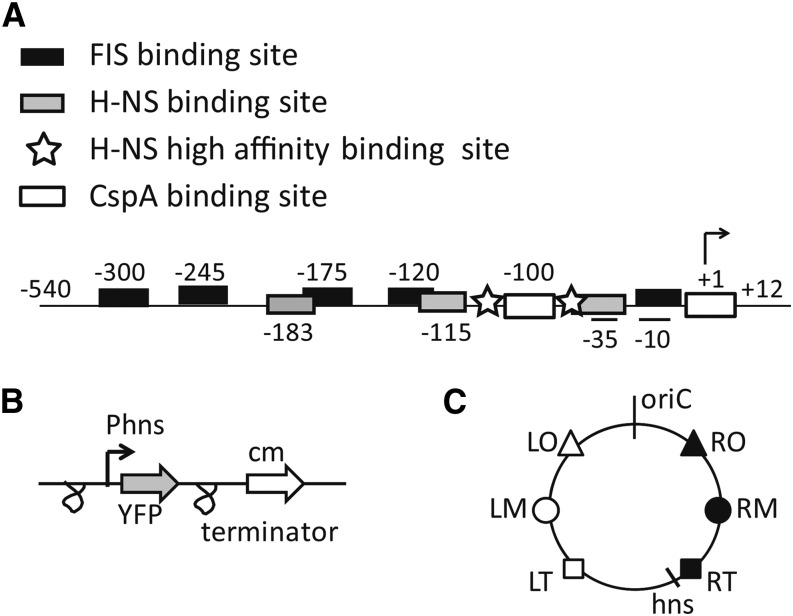
Schematic representation of the P*hns* promoter and of the insertions in the *E. coli* chromosome. (A) The boxes indicate the binding sites for different proteins in the P*hns* promoter region (black for FIS, gray for H-NS, white for CspA) as derived from the literature (La Teana *et al.* 1991; Ueguchi *et al.* 1993; Falconi *et al.* 1996). Stars indicate the H-NS high-affinity DNA binding sites (Lang *et al.* 2007). The -10, -35 regions and the transcription starting site, +1, also are annotated. (B) The promoter-*yfp* unit is flanked by two T1 terminators from the *E. coli rrnB* coding sequence. (C) Representation of the six different mirror sites on the *E. coli* chromosome in which the *yfp* gene was inserted under the control of the P*hns* promoter next to the gene conferring resistance to chloramphenicol. The symbols used here are the ones used to indicate these positions in Figure 2B, Figure 5 and Supporting Information, Figure S6. Details about the insertion positions can be found in Table 1. CspA, Cold shock protein A; FIS, factor for inversion stimulation; H-NS, histone-like nucleoid-structuring protein.

The reporter construction, comprising the P*hns* promoter upstream of the YFP gene next to an antibiotic resistance cassette, was inserted at six sites along the genome in three sets of mirror sites on each side of the origin of replication (Figure 1 and Table 1). Gene expression from the *hns* promoter is mainly regulated by FIS and the H-NS protein itself (Ueguchi *et al.* 1993; Falconi *et al.* 1993, 1996); therefore, these reporter strains can be used to measure the relative changes in activity of these two nucleoid proteins along the chromosome as a function of growth phase and growth rate. The strains containing the reporter construct in different positions were grown in a 96-well plate overnight to monitor the changes in OD and fluorescence as a function of time in growth media of different composition resulting in different growth rates. To control for the emergence of heterogeneity in the bacterial population, possibly leading to a decreased average amount of measured fluorescence from YFP, the amount of fluorescence per cell was also measured in parallel experiments by flow cytometry, for cells growing in exponential phase, in a flask, in a shaking incubator, confirming the results obtained in the plate reader (Supporting Information, Figure S1).

### The growth phase dependence of P*hns* promoter activity depends on the growth rate

**Figure 2:**
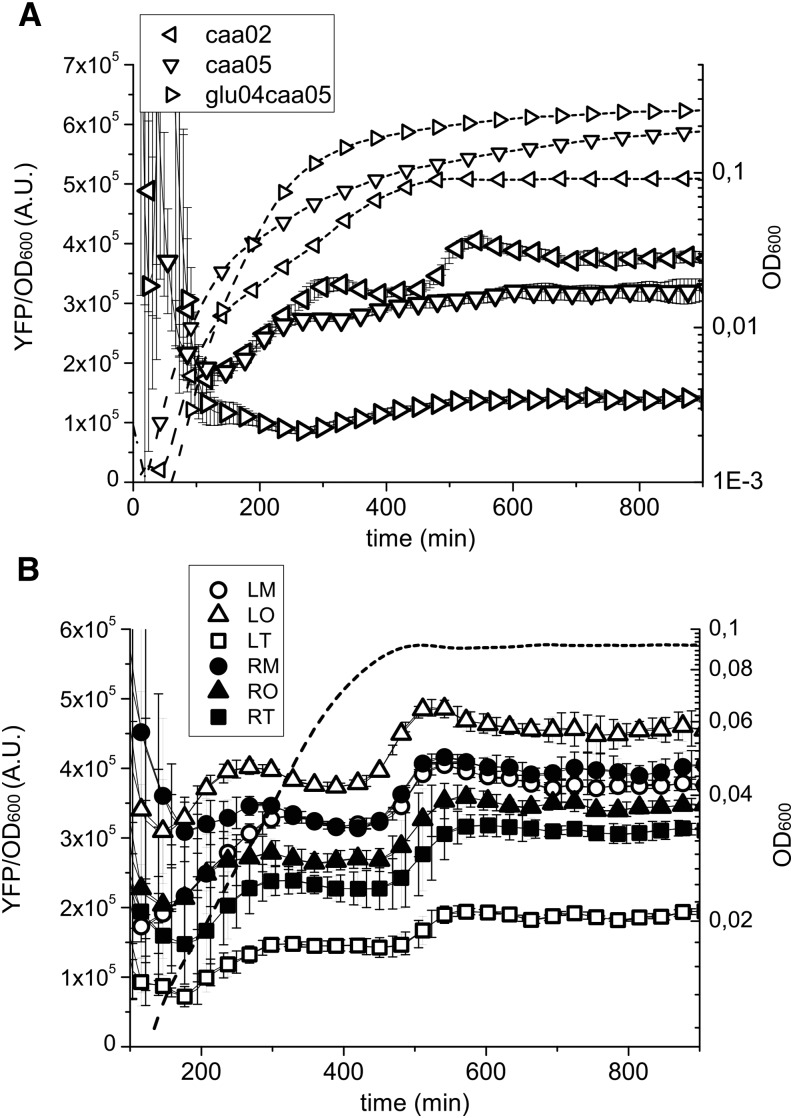
Change in OD and YFP concentration obtained from the plate reader measurements. The average protein concentration as a function of time is shown from technical triplicates within a single experiment, which is one of three independent experiments. The dashed lines represent the change in OD, whereas the continuous lines the change in YFP concentration. (A) The growth phase dependence of YFP concentration depends on the growth rate. At fast growth (glu04caa05), there is no accumulation of the protein while entering in stationary phase, whereas at slow growth (caa02), the amount of protein per unit of OD increases as cells approach to stationary phase. The data shown here are for the reporter in the LM position at 30°. This change in YFP concentration as a function of the growth medium doesn’t depend on the chromosomal position of the insertion. (B) YFP concentration depends on the chromosomal position of the gene. In the same growth conditions (caa02 at 30°), the amount of protein concentration is greater for insertions near the origin of replication (as expected due to gene copy number). However, in this growth medium the concentration is considerably lower for the strains with the insertion in the left terminus (LT, white squares) and right origin (RO, black triangles), than the ones in the right terminus (RT, black squares) and left origin (LO, white triangles), respectively, even though they are equally distant from the origin and thus with the same gene copy number. LM, left medium; LO, left origin; LT, left terminus; OD, optical density; RM, right medium; RO, right origin; RT, right terminus; YFP, yellow fluorescent protein.

The results obtained in the plate reader show that the change in YFP concentration as a function of growth phase depends on the growth rate (Figure 2A). In the richer medium (glu04caa05) the protein concentration is lower than in the other growth media (caa02 and caa05), and it remains more or less constant in the growth curve. In contrast, when the bacteria grow in the poorer media, there is an increase in protein concentration as the growth rate slows down during the transition to stationary phase. During growth in caa02, there is a second increase in concentration as the cells enter stationary phase. The high temporal resolution of the plate reader measurements allows us to obtain a measure of the change in promoter activity and growth rate as a function of time. These results indicate that during this growth phase transition, the growth rate slows down before the change in promoter activity does, resulting in a net accumulation of YFP (Figure S2). Therefore, a specific induction of *hns* promoter activity upon entry into stationary phase needs not to be invoked for this protein accumulation to take place. These results can provide an explanation for the different profiles in H-NS expression observed in previous studies (Ueguchi *et al.* 1993; Dersch *et al.* 1993; Free and Dorman 1995; Atlung and Ingmer 1997).

**Figure 3:**
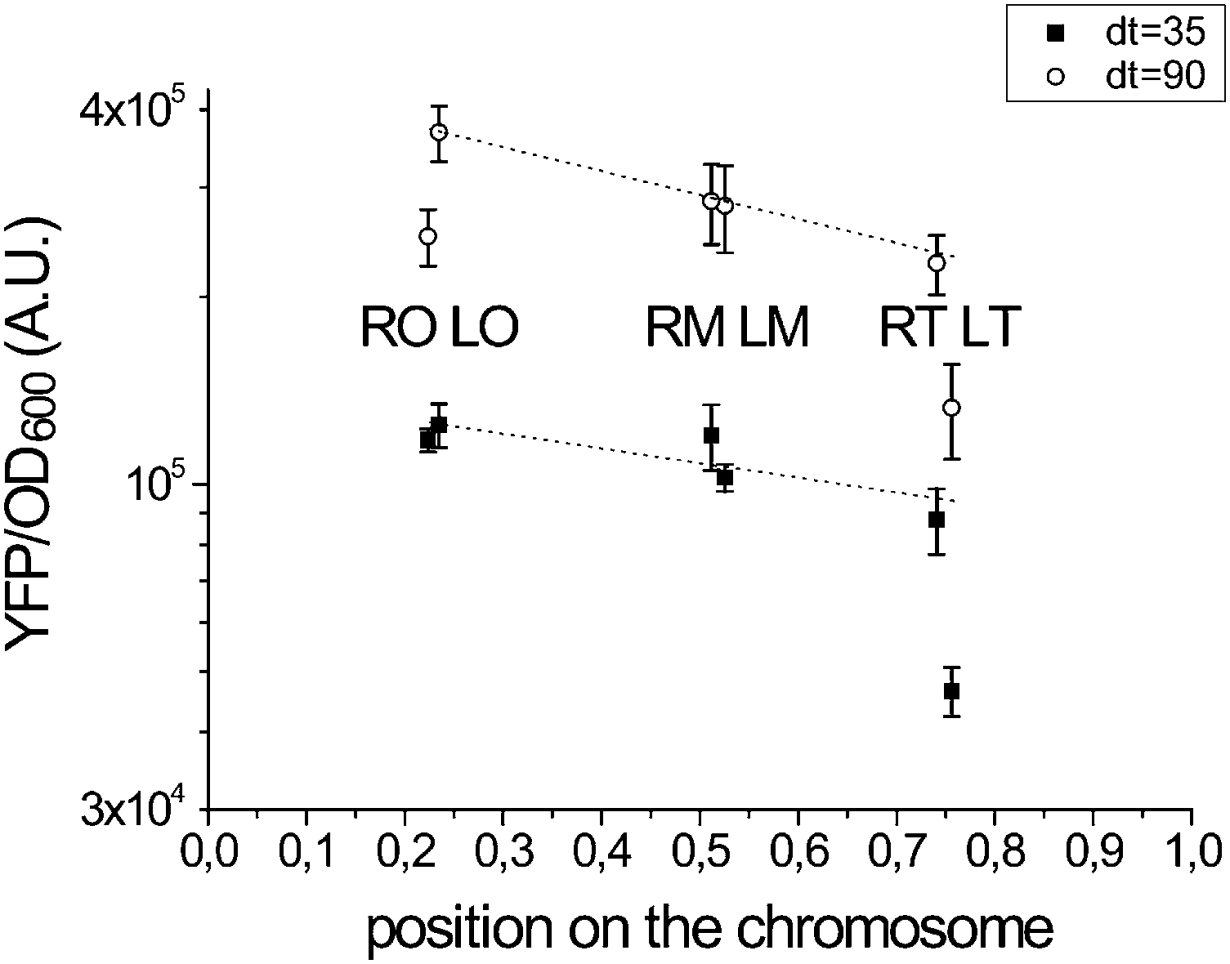
The difference in YFP concentration in exponential phase at two different growth rates can be explained for most positions by the change in gene copy number. YFP concentration was measured in mid exponential phase for the six chromosomal insertions at two different doubling times as a function of chromosomal position (0 for the origin of replication, 1 for the terminus). Data are the average of three independent plate reader experiments; the error bar corresponds to the SEM. The dotted line is the theoretical dependence of protein concentration as expected by the difference in gene copy number (Cooper-Helmstetter relation) for each growth rate (Cooper and Helmstetter 1968) (see the section *Materials and Methods*). The protein concentration for the LT strain is lower than what would be expected as a consequence of gene dosage, both at fast and at slow growth rates (glu04CAA05 and CAA02, respectively, at 30°). At slow growth, the concentration in the RO strain also deviates from the theoretical expectation. LM, left medium; LO, left origin; LT, left terminus; RM, right medium; RO, right origin; RT, right terminus; YFP, yellow fluorescent protein.

**Figure 4:**
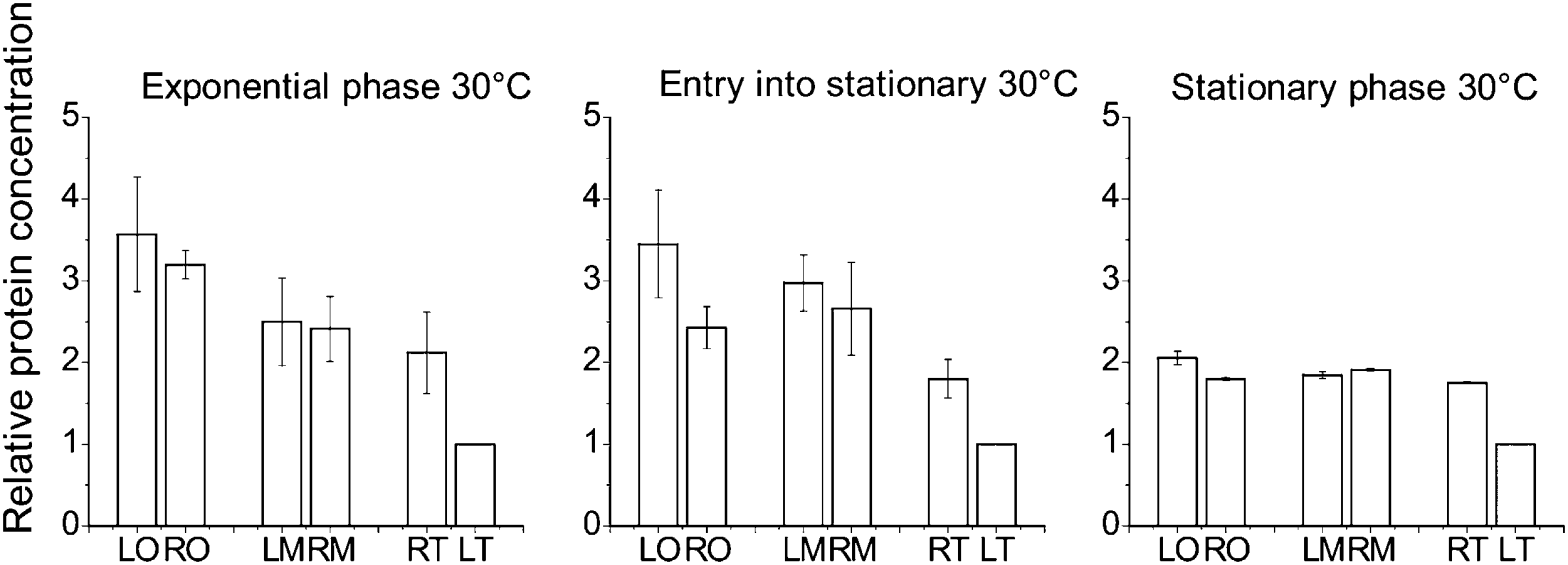
The difference in YFP concentration is larger at lower temperature and in the transition to stationary phase. The protein concentration was normalized by the LT values for strains in exponential, entry into stationary, and stationary phase (CAA05 at 30°) for three independent experiments, the error bar corresponds to the SEM. Data were taken at the time of maximum growth rate, at the time where the growth rate was half of the maximum and at growth rate equal to zero, respectively. The difference between YFP concentration in LO and RO strains arises at the entry into stationary phase. This is not observed when the experiment is carried out at 37° (see Figure S3). In stationary phase, the difference in protein concentration for all the positions decreases. The protein concentration for the LT strain is always lower than in the other strains. LM, left medium; LO, left origin; LT, left terminus; RM, right medium; RO, right origin; RT, right terminus; YFP, yellow fluorescent protein.

For most of the sites, comparison of YFP concentration as a function of genomic position in exponential phase shows a difference between the sites that can be explained by the differences in gene copy number expected from the DNA replication process (Figure 3). Interestingly, there is also a difference in gene expression between the sites that are equidistant from the origin, notably between left terminus (LT) and right terminus (RT) and right origin (RO) and left origin (LO). The latter difference becomes evident especially at slow growth and upon entry into stationary phase (Figure 3 and Figure 4).

### The differences in gene expression at positions equidistant from the origin increase at slower growth rates

**Figure 5:**
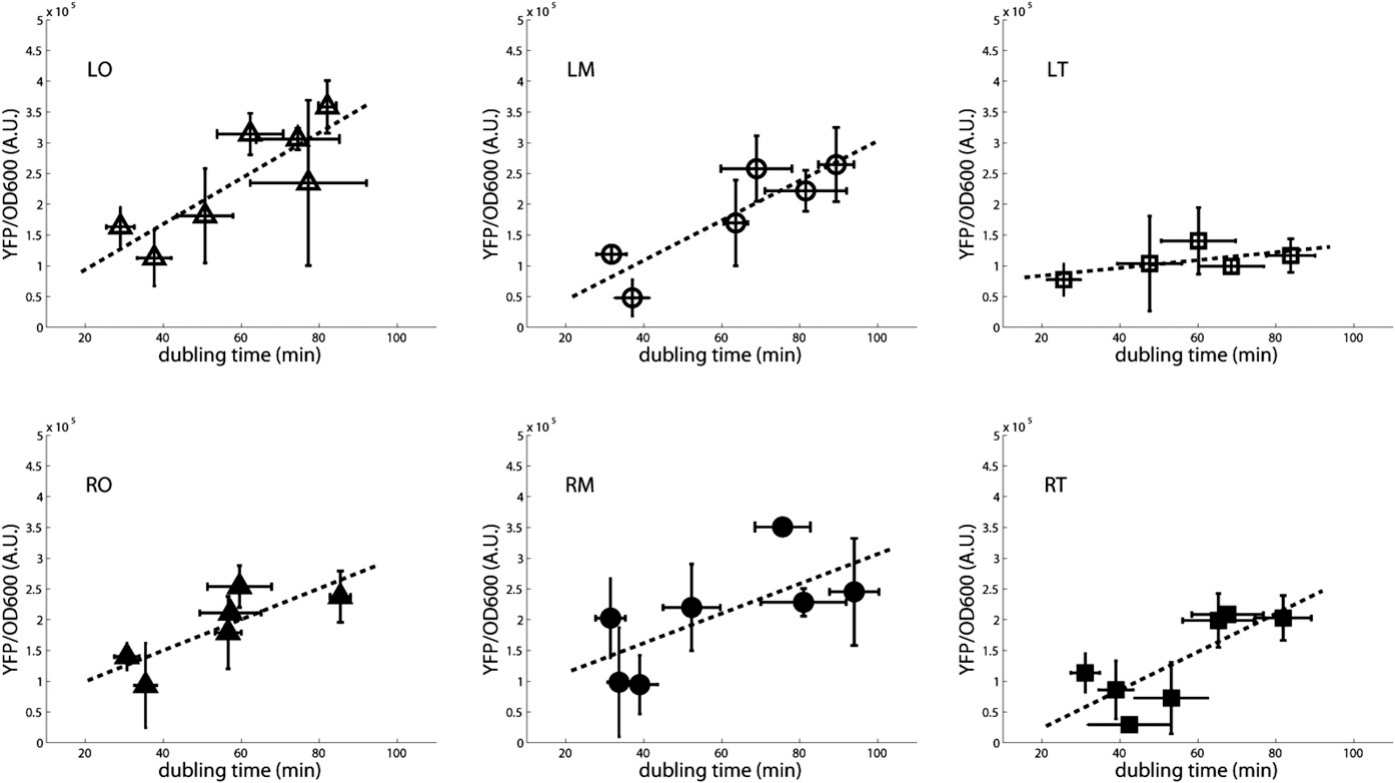
The change in YFP concentration as a function of growth rate shows an increase in protein dilution at faster growth and a growth rate dependence in the difference between RT and LT. Protein concentration for samples growing in M9 minimal media supplemented with various concentration of glucose and casamino acids at 30°. The error bars are the SEM resulting from three independent experiments. Data were taken at mid exponential phase. At fast growth there is a low YFP concentration due to a faster dilution rate. For the LT strain (white squares) the YFP concentration values are low also at slow growth, indicating a stronger repression in this position than at the others. LM, left medium; LO, left origin; LT, left terminus; RM, right medium; RO, right origin; RT, right terminus; YFP, yellow fluorescent protein.

By measuring the amount of fluorescence in exponential phase in the different growth media, these experiments show a decrease in YFP concentration as a function of increasing growth rate (Figure 5). The faster dilution rate at faster growth results in a lower YFP concentration. This finding is observed for all six positions, independently of the expected increase in copy number due to DNA replication of the sites near the origin. Figure 3 shows in fact that the change in copy number can explain the differences in gene expression between the different positions at the different growth rates. This finding is consistent with all positions having the same promoter activity that in addition does not change significantly with growth rate. The activation of the P*hns* promoter by FIS in exponential phase at fast growth (Falconi *et al.* 1996) doesn’t seem sufficient to counteract the dilution rate. There is, however, one strain that does not follow this trend, the one in which the reporter is inserted at the LT position. In this case, the concentration of YFP remains almost constant as a function of growth rate.

### H-NS binding density due to greater AT content correlates with promoter repression

**Figure 6:**
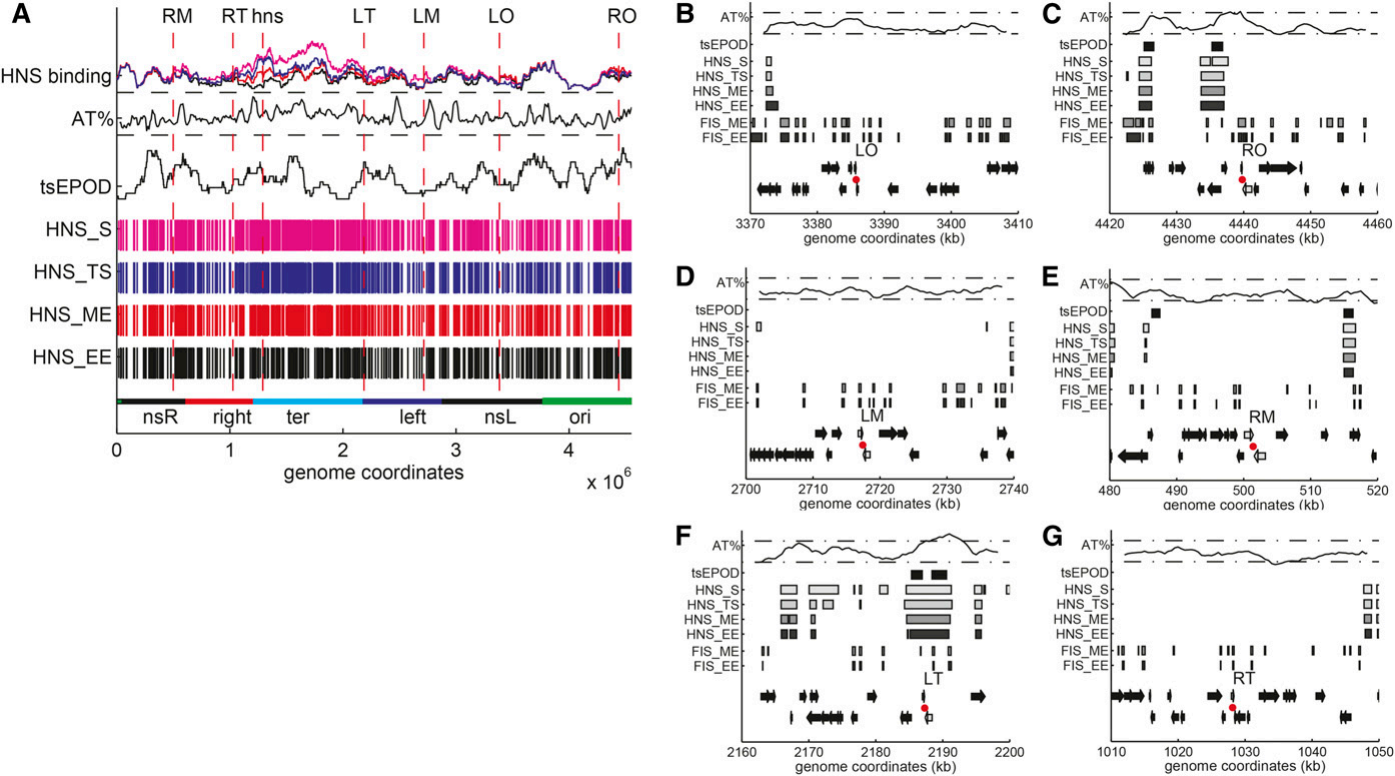
(A) Global view of H-NS binding, AT content, and presence of tsEPODs in the *E. coli* chromosome. From the bottom to the top: The macrodomains, as defined by Boccard (Valens *et al.* 2004). Sites bound by H-NS in early exponential (HNS_EE), mid exponential (HNS_ME), transition to stationary (HNS_TS), and stationary phase (HNS_S) (Kahramanoglou *et al.* 2011). There is an increase in the number and the length of regions bound by H-NS when approaching stationary phase, especially in the terminus. tsEPODs mapped on the *E. coli* chromosome (Vora *et al.* 2009). The plot shows the number of genes overlapping with tsEPODs as determined from the NUST software, with multiple sliding windows histogram performed choosing a bin number equal to 32 (Scolari *et al.* 2012). The AT content is calculated with a sliding window of 50 kb with a shift of 10 kb. The two horizontal dashed lines correspond to 45 and 55% AT. The terminus shows greater AT-content. H-NS binding: sites bound by H-NS in the different growth phases (Kahramanoglou *et al.* 2011). (B−G) Genomic neighborhood of the chromosomal insertion positions of the reporter construct. From the bottom to the top of each plot: Genes on the lagging and on the leading strand. In gray, the two convergent genes between which the P*hns*-yfp construct was inserted. The site of insertion is shown by a red dot. Position of the sites bound by FIS in early exponential (FIS_EE) and in mid exponential (FIS_ME) phases (Kahramanoglou *et al.* 2011). There are more sites bound by FIS near the origin (LO and RO, plots on the top) than in the terminus (LT and RT, plots in the bottom). Position of the sites bound by H-NS in early exponential (HNS_EE), mid exponential (HNS_ME), transition to stationary (HNS_TS) and stationary (HNS_S) phases (Kahramanoglou *et al.* 2011). In the proximity of LT and RO there is an extended region of sites bound by H-NS. Presence of genes identified as transcriptionally silent (Vora *et al.* 2009). The AT content is calculated with a 4000-bp sliding window with a shift of 500 bases. The two horizontal dashed lines correspond to 45 and 55% AT. A peak in AT-content is visible near LT and RO. FIS, factor for inversion stimulation; H-NS, histone-like nucleoid-structuring protein; LM, left medium; LO, left origin; LT, left terminus; RM, right medium; RO, right origin; RT, right terminus; tsEPODs, transcriptionally silent extended protein occupancy domains.

The activity of the P*hns* promoter depends on the activation by the FIS protein in early exponential phase at fast growth (Falconi *et al.* 1996), Cold shock protein A (CspA) for induction upon cold shock (La Teana *et al.* 1991), and on the binding of the H-NS protein itself resulting in repression (Ueguchi *et al.* 1993; Falconi *et al.* 1993). Binding of the H-NS protein along the genome is not uniform and changes as a function of the growth phase (Kahramanoglou *et al.* 2011; Zarei *et al.* 2013). When the sites of insertion of the reporter construct are mapped on the H-NS binding patterns one can see that those sites that are less expressed (LT and RO) are found in regions with a greater probability of H-NS binding as measured by formaldehyde crosslinking (Figure 6 and Figure S4).

Furthermore, it has been shown that the binding of H-NS to the lower-affinity sites leading to oligomerization and repression is temperature dependent (Bouffartigues *et al.* 2007). Comparing the results obtained at 30° and 37°, one can see that the differences between LT and RT and RO and LO are greater as the temperature is decreased, again pointing to an H-NS dependent effect (compare Figure 4 and Figure S3). Finally, the loss of the difference between the expression in RT and LT strains in bacteria lacking H-NS confirms that the reduced expression of LT is due to an increased level of repression by H-NS (Figure S5).

To test whether the difference in expression was specific to the location of the original insertion sites, additional strains were created with insertions at two distances from LT, at about 18000 and 135000 base pairs (LT1 and LT2 respectively) and one additional strain with an insertion at about 66000 base pairs from RT (RT1). The reporters at these sites exhibit a similar level of expression as the original sites (Figure S6), indicating that the differences observed between LT and RT are not limited to the local genomic environment, or to the presence of tsEPODs at the LT insertion site (see paragraph below), but that H-NS activity can affect sets of genes within a larger region of the genome. If one takes into account the position of the H-NS binding sites with respect to the location of the reporter gene (Figure S4) one can see however that RT1 is found in an H-NS−rich region compared with RT (Figure 6, B−G): this results in increased repression upon entry into stationary phase ( Figure S6), similarly to what is observed when comparing RO with LO (Figure 4).

Previous work had identified regions of the *E. coli* genome that are rich in DNA-bound proteins and have a lower average level of expression compared with other genes, called tsEPODs, for transcriptionally silent Extensive Protein Occupancy Domains (Vora *et al.* 2009). A subsequent analysis of the correlation of the genes within these regions with H-NS binding confirmed the colocalization of significant clusters of both tsEPODs and H-NS binding, particularly in regions containing horizontally acquired genes, suggesting that tsEPOD could correspond to H-NS−rich regions (Zarei *et al.* 2013). To determine whether any of the six positions chosen here for the insertion of the reporter constructs is found within such a genomic region, the sites of insertions were placed within a genome map summarizing the previously published results obtained on the location of tsEPODs, H-NS, and FIS occupancy at different stages of the growth curve next to the AT content (Figure 6 and Figure S4). This analysis shows that some of the reporter sites overlap with both tsEPODs and H-NS binding along the genome. However, the decreased level of expression correlates better with H-NS binding regions than with the presence of tsEPODs, as described for example for the result obtained comparing the reporter at LT with the ones at LT1 and LT2.

### Moving the *hns* gene itself does not have a significant effect on the cell’s phenotype or fitness in the short term

The results obtained with the reporter strains naturally lead to the question of whether the position of the *hns* gene near the terminus has been conserved through evolution because it results in a specific advantage in growth and thus an increase in fitness. To test this the *hns* gene was moved to the six genomic positions used here, we then tested the ability of these strains to survive the acid and cold shocks that are a hallmark of H-NS activity (Genet *et al.* 1994; Giangrossi *et al.* 2005) or their ability to compete with the wild-type strain in different growth conditions. In addition, because the Δ*hns* strain is known to lose its ability to swarm (Soutourina *et al.* 1999), we also carried out swarming assays. All of these experiments showed that the mutant strains grew equally well compared with the wild type (data not shown). This might be due to the fact that, in addition to being a negatively autoregulated gene, H-NS expression is also regulated posttranscriptionally by the DsrA RNA resulting in a robust expression level of the protein independently from its local genomic environment (Lease and Belfort 2000). Moreover, H-NS activity is closely coupled with its interaction with other proteins, such as StpA and Hha that can complement its activity (Zhang *et al.* 1996; Williams *et al.* 1996; Sonnenfield *et al.* 2001; Paytubi *et al.* 2004; Madrid *et al.* 2007; Ueda *et al.* 2013). Displacing the *hns* gene therefore does not have a significant effect, at least in the timescales of a few days probed of our experiments. It is, however, still possible that there might be an effect on a longer-term evolution experiment (Chib and Mahadevan 2012). We are currently exploring this possibility.

## Discussion

### Measuring changes in a transcription regulator activity *in vivo*: heterogeneous effects along the genome

In the current work, we provide evidence for an uneven effect of H-NS dependent regulation of a reporter gene expression along the genome of *E. coli*. Furthermore we have shown that these differences depend on the growth conditions, such as growth phase, growth rate, and temperature, reflecting changes in H-NS activity along the genome and thus also changes in nucleoid structure and organization as the cell adapts to different growth environments.

Because the P*hns* promoter is known to be repressed by H-NS itself via the presence of specific H-NS binding sites and AT-rich sequences upstream of the core promoter region (Ueguchi *et al.* 1993; Falconi *et al.* 1993; Giangrossi *et al.* 2001; Lang *et al.* 2007), the promoter-reporter construct used here measures the local activity of H-NS. The high affinity of H-NS for AT-rich sequences can result in a greater local concentration of the protein in AT-rich regions of the nucleoid, such as the terminus, and in a greater level of DNA binding cooperativity (Azam and Ishihama 1999). For example, H-NS is known for playing an important role in silencing horizontally acquired genes, which tend to be more AT-rich than the rest of the genome, and which sometimes also include pathogenicity islands (Dorman 2013). This level of repression takes place most of the time, except when the genes of the pathogenicity island are induced by specific environmental changes coupled with the activity of a transcription factor (Beloin *et al.* 2002; Prosseda *et al.* 2004; Kane and Dorman 2011). Partly because of the acquisition of heterologous DNA fragments, the AT content along the genome is not equally repartitioned, with regions as long as tens of kilobases having a greater average AT content than surrounding sequences (Lawrence and Ochman 1997; Eisen 2000) (Figure 6).

### Three different levels of H-NS–dependent regulation as a function of chromosomal position

We found that P*hns* promoter expression in the LT position is always more repressed by H-NS than the other sites, such as RT, which is equidistant from the origin. The reporter at the LT position, and its neighboring insertions, LT1 and LT2, are situated in a ~10-kb region with an AT content greater than average (Figure 5 and Figure S4). The repression in LT is probably enhanced by the fact that it is found near the boundary with the left macrodomain, featuring a significant cluster of horizontally acquired AT-rich genes and silent pseudogenes (Zarei *et al.* 2013).

The greatest difference between the LT and RT sites is observed when looking at the growth rate dependence of expression. LT is significantly more repressed than RT at slower growth rates, resulting in a constant YFP concentration as a function of doubling time (Figure 5). This finding indicates that H-NS activity is greater at the LT site at slow growth. A similar effect is observed comparing RO and LO, but to a smaller extent (Figure 3). The constant YFP concentration (YFP/OD equivalent to YFP/mass) as a function of growth rate of the LT strain is consistent with what would be expected from a gene cooperatively repressed by negative auto-regulation (Klumpp *et al.* 2009). The *hns* gene is found in an environment similar to the one of LT (Figure S4), therefore this suggests that H-NS itself may follow a similar pattern of expression. At slower growth, there is a reduced dilution rate and a decreased amount of DNA per cell and thus less H-NS binding sites per cell. This could result in a greater amount of H-NS available to bind a promoter and repress gene expression, particularly in the case of decreasing amounts of FIS (see paragraph below). The other strains are less sensitive to H-NS repression in exponential phase. Variation of their YFP concentration as a function of growth rate is similar to that expected either from a weakly repressed or a constitutive promoter, whose gene product becomes more diluted at fast growth, independently of the gene copy number.

A different pattern in the level of expression is observed when the growth rate slows down during entry into stationary phase. During this growth phase transition the YFP concentration from RO is significantly lower than the one measured in LO. Similarly, in these conditions the expression from RT1 is lower than the one from RT. An important factor contributing to the difference in expression among the strains can be found in the interplay between the NAPs along the bacterial growth curve. In the literature, there is ample evidence for changes in the composition of NAPs according to growth phase and to growth rate of the cell (Ishihama 1999; Azam *et al.* 1999; Ohniwa *et al.* 2006; Dillon and Dorman 2010; Browning *et al.* 2010). FIS, a NAP known for being necessary for fast growth (Nilsson *et al.* 1992a) and for being involved in shaping the chromosome (Schneider *et al.* 2001; Dame 2005), is expressed in a growth rate and growth phase dependent fashion. A peak of the cellular FIS concentration is observed in exponential phase, while it becomes undetectable in stationary phase (Nilsson *et al.* 1992a; Azam *et al.* 1999). In exponential phase, the greater FIS concentration can better compete with H-NS at the P*hns* promoter (Falconi *et al.* 1996), explaining the lack of difference in expression between the sites during this growth phase.

The competition between FIS activation and H-NS repression occurs also at other genes, such as the ribosomal promoters (Nilsson *et al.* 1992b; Afflerbach *et al.* 1998; Schröder and Wagner 2000). Furthermore, in similar growth conditions as those used here, chromatin immunoprecipitation assays have shown that there is a significant overlap between FIS binding and AT-rich regions and FIS and H-NS binding to the genome (Grainger *et al.* 2006; Cho *et al.* 2008). Therefore, when the amount of FIS in the cell decreases at the end of exponential phase, H-NS can extend its action on the chromosome and to a greater extent in the regions with a greater amount of H-NS binding sites. Our data support this idea: the expression from LO and RO is similar when FIS is abundant, and lower in RO when FIS concentration decreases, due to the presence of AT-rich H-NS binding sites near RO. The same effect can be seen in the difference between RT and RT1 at entry into stationary phase.

Recent results obtained on purified nucleoids have shown that H-NS plays an important role in maintaining the level of compaction of the nucleoid when the total level of transcription activity decreases during the transition to stationary phase (Thacker *et al.* 2014). Previous studies have shown that ongoing transcriptional activity can contribute to increased compaction of the nucleoid (Cabrera *et al.* 2009; Jin *et al.* 2013). Most of the transcriptional activity in the cell derives from ribosomal genes that are activated by FIS in exponential phase and repressed by H-NS (Afflerbach *et al.* 1998; Schröder and Wagner 2000). An increase in H-NS to DNA ratio upon entry into stationary phase could thus explain at the same time both the decrease in total transcription activity and the compensatory compaction of the nucleoid. This H-NS−dependent repression, however, takes place first at the genes within AT-rich regions of the nucleoid during the transition to stationary phase and then is extended to the other genes as cell growth slows down even more in stationary phase.

In a parallel work, similar reporter constructs using the *fis* and *dps* genes promoters were inserted in some of the same positions used here (Berger, M., V. Gerganova, U. Dobrindt, A. Travers, and G. Muskhelishvili, unpublished data). Preliminary data from both of these reporter constructs shows a chromosomal position−dependent expression, but only in a *hupA/B*- background, in which the global nucleoid structure is altered (Jaffe *et al.* 1997). For the *dps* promoter a chromosomal position dependent expression was observed when the insertion was placed at the LT position or within a specific genomic island in the ABU8379 strain, known for being AT-rich and for containing nonessential genes (Hacker and Kaper 2000). The *dps* promoter is also regulated by both H-NS and FIS, albeit by a different mechanism compared to the P*hns* promoter that results in its induction in late stationary phase (Grainger *et al.* 2008). These results therefore support the proposal that H-NS regulation is necessary but not sufficient to determine a chromosomal position dependence of gene expression and that this positional effect is also dependent on the promoter sequence used.

Recently, a similar analysis of gene expression as a function of chromosomal position has been presented by Block *et al.* (2012) and Ying *et al.* (2014). They inserted in different positions of the chromosome the gene for a fluorescent reporter protein under the control of a synthetic promoter, P*Lac*O-1 or P*tet*, respectively. The first is an inducible promoter repressed by LacI, whereas the second is repressed by TetR. In agreement with previous results obtained in *E. coli* and other bacterial species (Chandler and Pritchard 1975; Schmid and Roth 1987; Sousa *et al.* 1997; Thompson and Gasson 2001; Dryselius *et al.* 2008), no differences in expression as a function of chromosomal position, gene orientation and regulator-target gene distance was detected. A possible reason for the differences between our results and the ones described above could lie in the different promoters used, since the promoter used here is repressed specifically by H-NS. Furthermore in this case we carried out measurements as a function of growth phase and growth rate while in most of the works cited above gene expression was measured in mid exponential phase in a single or at most two growth media. On the other hand, the result showing that the concentration of YFP expressed from *Phns* at the different chromosomal insertions doesn’t depend on the distance from the regulator gene agrees with the data obtained previously.

More recently, Bryant *et al.* (2015) have observed for the first time an effect of chromosomal position on the expression of a GFP reporter under control of the *lac* promoter. Their study revealed a change in expression due to the transcription activity of neighboring genes and an effect of negative supercoiling in the induction of the reporter’s expression at specific sites along the genome. They also observed a decrease in gene expression when the reporter was inserted within a tsEPOD, which has allowed them to propose that tsEPOD are not necessarily composed of poorly expressed genes but constitute silenced regions of the genome. The insertion of the reporter cassette at other sites also resulted in decreased expression which however was not correlated with the presence of a tsEPOD. This agrees with the results obtained here, which show that tsEPOD do correlate with the level of expression in some cases, but that the AT content is more important in the specific case of a promoter regulated by nucleoid proteins.

A number of cellular parameters affecting the gene expression change during both growth transitions and cellular adaptation. These include the increased dilution rate of transcription factors due to the cell division and the amount of DNA per cell due to overlapping DNA replication rounds; the amount of active ribosomes and of available RNA polymerase, taking into account changes in the amounts of the different sigma factors; the concentration of small metabolites, such as ppGpp and cAMP; the concentration of nucleoid proteins and the level of negative supercoiling (Travers and Muskhelishvili 2005; Berthoumieux *et al.* 2013; Klumpp and Hwa 2014). This type of regulation can be thought of as an analog control, complementing the digital control, *i.e.*, the network of regulation mediated by dedicated transcription factors (Blot *et al.* 2006; Marr *et al.* 2008; Sonnenschein *et al.* 2011). All of these factors can potentially affect gene expression independently of where the genes are found in the genome. The genomic sequence, however, and especially its AT content, can affect both the affinity for nucleoid proteins and the stability of the DNA under torsional stress due to changes in topology (Sobetzko *et al.* 2013).

Here, we provide evidence for modulation of gene expression depending on the chromosomal position by a global regulator. We have identified three different levels of regulation: those regions where H-NS has small effect (RT, LO, RM, LM); regions in which regulation by H-NS is conditional (RT1 and RO) and a region in which H-NS repression is strongest and results in a growth rate−independent protein concentration (LT and its neighbors, LT1 and LT2). This indicates that the position of H-NS regulated genes will influence their growth phase, growth rate, and temperature dependence of expression. The nucleoid protein−dependent structure of the chromosome can thus affect the gene expression in *E. coli*, it would now be interesting to extend this approach to other nucleoid proteins and other bacterial species in order to test the generality of these conclusions.

## 

## Supplementary Material

Supporting Information
