# Supplementary material for: Gene Regulation by H-NS as a Function of Growth Conditions Depends on Chromosomal Position in Escherichia coli
Source: G3 (Bethesda). 2015 Feb 19;5(4):605–14. doi: 10.1534/g3.114.016139 (PMC4390576; doi:10.1534/g3.114.016139)
Supplement: Supporting Information [file supp_5_4_605__index.html]

Gene Regulation by H-NS as a Function of Growth Conditions Depends on Chromosomal Position in Escherichia coli — Supporting Information 

# Gene Regulation by H-NS as a Function of Growth Conditions Depends on Chromosomal Position in *Escherichia coli*

## Supporting Information for Brambilla and Sclavi, 2015

**Files in this Data Supplement:**

- Supporting Information - Figures S1-S6 (PDF, 406 KB)
- Figure S1 - Fluorescence intensity within the bacterial population is homogeneous. (PDF, 110 KB)
- Figure S2 - The increase in YFP concentration in stationary phase at slow growth (CAA02) is due to the delay in the decrease of promoter activity with respect to the decrease in growth rate. (PDF, 115 KB)
- Figure S3 - No difference in YFP concentration between RO and LO as a function of growth phase at 37°C compared to 30°C (Fig. 4 in the main text). (PDF, 95 KB)
- Figure S4 - Genomic neighborhood of the additional insertions near LT and RT and of the *hns* gene shows that these sites are found in regions with a higher than average AT content and large H-NS bound regions that increase in size as the cells enter stationary phase. (PDF, 252 KB)
- Figure S5 - The difference between RT and LT is lost in a ∆*hns* background. (PDF, 108 KB)
- Figure S6 - P*hns* promoter activity remains similar for insertions placed up to 135 Kb away from the original sites. (PDF, 124 KB)
