# Supplementary material for: Gene Regulation by H-NS as a Function of Growth Conditions Depends on Chromosomal Position in Escherichia coli
Source: G3 (Bethesda). 2015 Feb 19;5(4):605–14. doi: 10.1534/g3.114.016139 (PMC4390576; doi:10.1534/g3.114.016139)
Supplement: Supporting Information [file supp_g3.114.016139_016139SI.pdf]

G3 · Genes | Genomes | Genetics

Gene regulation by H-NS as a function of growth conditions depends on chromosomal position in *E. coli*.

Elisa Brambilla[§,†] , Bianca Sclavi[§1]

§ LBPA, UMR 8113 du CNRS, Ecole Normale Supérieure de Cachan, 61 Avenue du Président Wilson, 94235, Cachan, France

† School of Engineering and Science, Jacobs University Bremen, Campus Ring 1, 28759 Bremen, Germany

1 Corresponding Author: LBPA, UMR 8113 du CNRS, Ecole Normale Supérieure de Cachan, 94235, Cachan, France. E-mail :

sclavi@lbpa.ens-cachan.fr

DOI 10.1534/g3.114.016139

Supporting Information

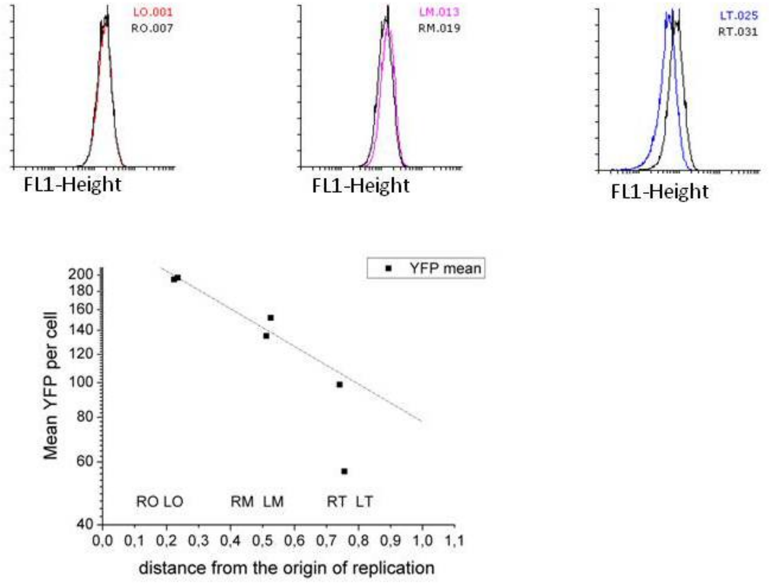

Figure S1 Fluorescence intensity within the bacterial population is homogeneous. Example of FACS measurement of the six bacterial strains. The distribution of YFP per cell is normal for all the strains, excluding therefore heterogeneity in the population. In the plot comparing LT and RT it is possible to notice how the LT strain (blue) is less fluorescent than the RT strain (black). This is more evident when plotting the mean of the YFP distribution as a function of the distance from the origin of replication. The mean YFP for the LT strain is lower than that expected from the difference in gene copy number (dotted line).

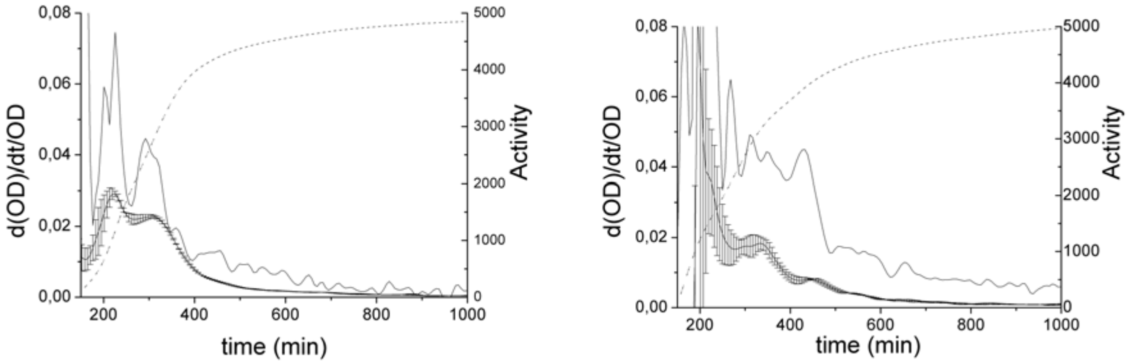

A

B

Figure S2 The increase in YFP concentration in stationary phase at slow growth (CAA02) is due to the delay in the decrease of promoter activity with respect to the decrease in growth rate. The change in OD is shown by a dashed line, the change in growth rate (d(OD)/dt/OD) in the line with the error bars from the three technical repeats within the experiment and the change in promoter activity (d(YFP)/dt/OD) by a continuous line. (A) Growth rate and promoter activity for cells growing in M9 minimal media supplemented with 0.4% glucose and 0.5% casamino acids (fast growth). The decrease in growth rate at the entry into stationary phase happens at the same time as the decrease in promoter activity, leading to no accumulation of YFP (Fig. 2A main text). (B) Growth rate and promoter activity for cells growing in M9 minimal media supplemented with 0.2% casamino acids (slow growth). The decrease in promoter activity takes place later than the decrease in growth rate, there is therefore an accumulation of YFP at the entry into stationary phase for cells growing in a poorer medium.

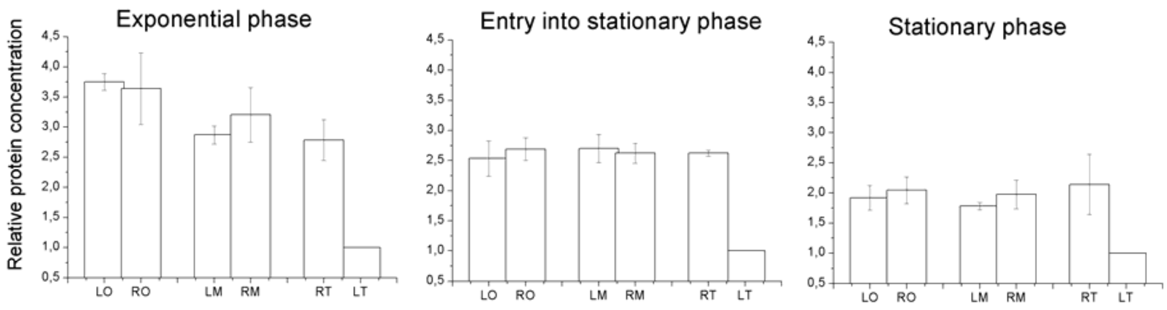

Figure S3 No difference in YFP concentration between RO and LO as a function of growth phase at 37°C compared to 30°C (Fig. 4 in the main text). YFP concentration was normalized by the LT values for strains in exponential, entry into stationary and stationary phase for three independent experiments, the error bars indicate the SEM. Data were taken at the time of maximum growth rate, at the time where the growth rate was half of the maximum and at growth rate equal to zero, respectively. The YFP concentration in the LT strain is always lower than in the other strains. AT 37°C there is no significant difference between LO and RO.

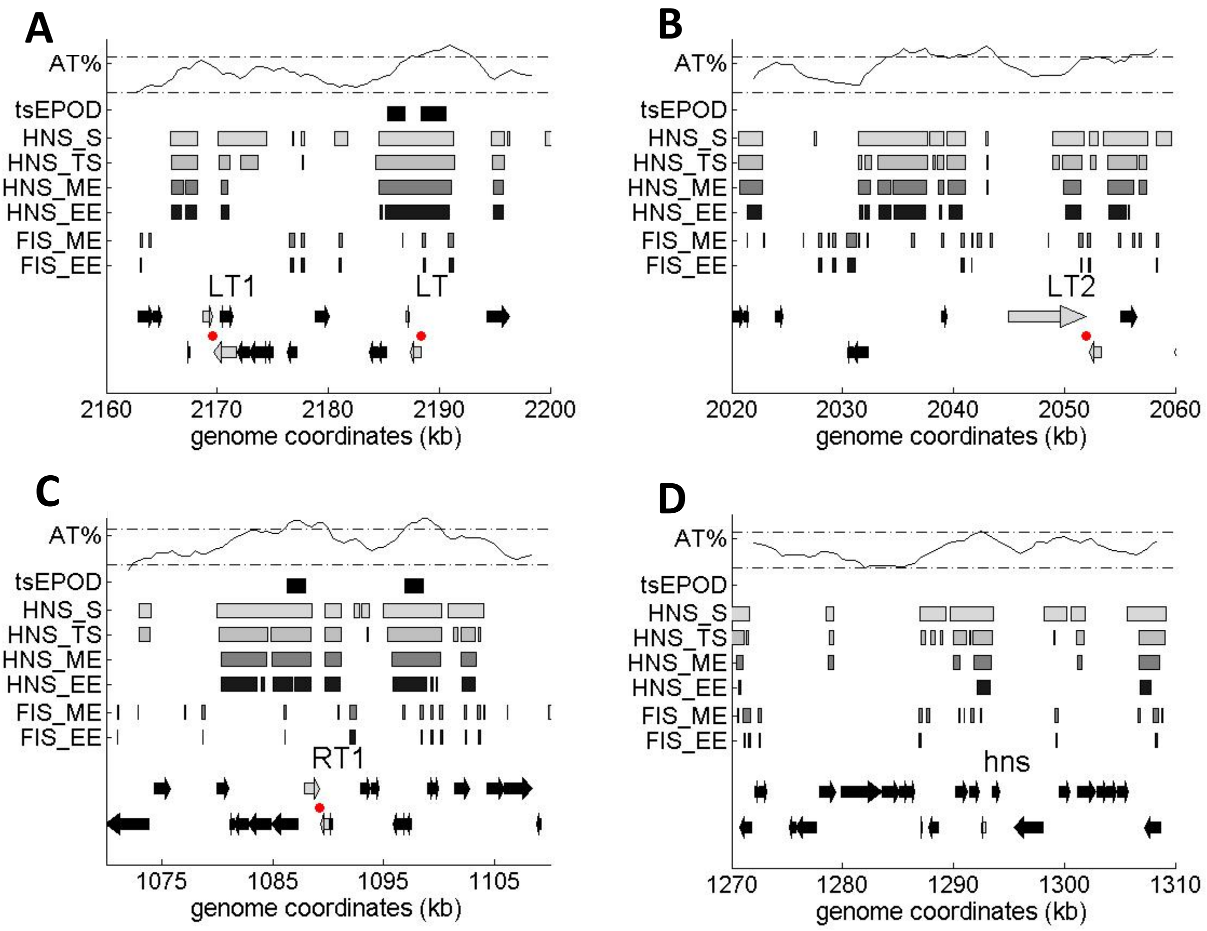

Figure S4 Genomic neighborhood of the additional insertions near LT and RT and of the *hns* gene shows that these sites are found in regions with a higher than average AT content and large H-NS bound regions that increase in size as the cells enter stationary phase. For each plot, from the bottom to the top**:** Genes on the lagging and on the leading strands. In grey are plotted the convergent genes where the insertions have been made, and the *hns* gene itself. FIS binding in early exponential phase (FIS_EE) and in mid-exponential phase (FIS_ME), data from (Kahramanoglou *et al.* 2011). Sites bound by H-NS in early exponential (HNS_EE), mid-exponential (HNS_ME), transition to stationary (HNS_TS) and stationary (HNS_S) phases (Kahramanoglou *et al.* 2011). On the top of these, in LT and in RT1 there are also tsEPOD (Vora *et al.* 2009). AT content is calculated within a 4000 bp sliding window with a shift of 500 bases. In the proximity of all the insertions it is possible to detect a strong occupancy by H-NS as well as a higher AT content of the genomic sequence. For the *hns* gene there is a clear increase in the length of the H-NS bound region while cells are passing from exponential to stationary phase.

Additional references:

Kahramanoglou, C., A. S. N. Seshasayee, A. I. Prieto, D. Ibberson, S. Schmidt *et al.*, 2011 Direct and indirect effects of H-NS and Fis on global gene expression control in Escherichia coli. Nucleic Acids Res. 39: 2073–2091.

Vora, T., A. K. Hottes, and S. Tavazoie, 2009 Protein Occupancy Landscape of a Bacterial Genome. Mol. Cell 35: 247–253.

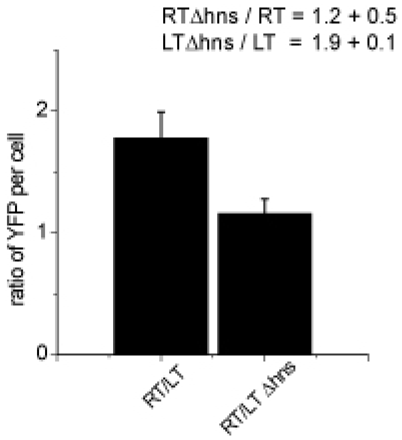

Figure S5 The difference between RT and LT is lost in a Δ*hns* background. The bar graph shows the ratio between the average YFP per cell for the RT and LT insertions in a WT background and Δ*hns* background. Data were obtained by flow cytometry for cells in exponential phase growing in 0.5% casamino acids at 37°C in flasks, shaking. Error bars represent the standard deviation for four independent experiments. The ratio between the fluorescence in RT and LT becomes closer to 1 in the Δ*hns* background with respect to the ratio in the wild type background. This is due to a stronger increase in fluorescence for the LT position in absence of H-NS with respect to the slight increase of fluorescence for RT position in the Δ*hns* background.

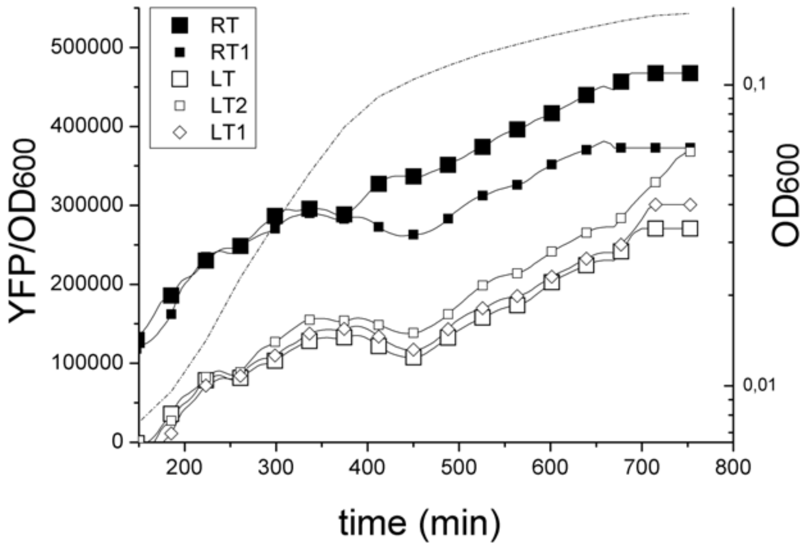

Figure S6 P*hns* promoter activity remains similar for insertions placed up to 135 Kb away from the original sites. Plate reader experiment for strains growing in M9 supplemented with 0.2% casamino acids, at 37°C. The effect of silencing in the LT position extends over several tens of kilobases (LT position= 2185402, LT2=2050038 (Δ135364), LT1=2167635 (Δ17767)), such that the signal from the neighboring strains LT1 and LT2 is similar to the one in LT strain. The YFP concentration in RT1 is similar to the one in RT (RT=1027582, RT1=1093457 (Δ65875)) during exponential phase, while at the entry into stationary phase the expression of RT1 is lower than that of RT.
