# Supplementary material for: Gene Regulation by H-NS as a Function of Growth Conditions Depends on Chromosomal Position in Escherichia coli
Source: G3 (Bethesda). 2015 Feb 19;5(4):605–14. doi: 10.1534/g3.114.016139 (PMC4390576; doi:10.1534/g3.114.016139)
Supplement: Supporting Information [file supp_g3.114.016139_FigureS1.pdf]

Supporting Information

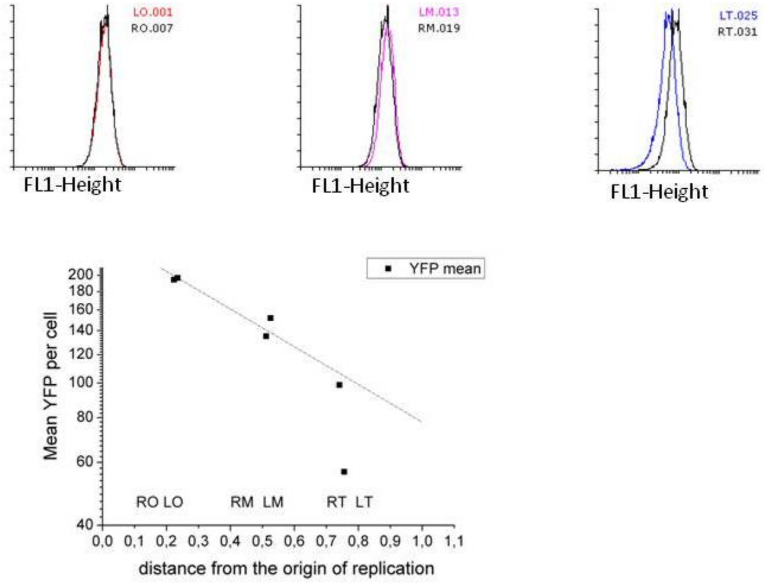

Figure S1 Fluorescence intensity within the bacterial population is homogeneous. Example of FACS measurement of the six bacterial strains. The distribution of YFP per cell is normal for all the strains, excluding therefore heterogeneity in the population. In the plot comparing LT and RT it is possible to notice how the LT strain (blue) is less fluorescent than the RT strain (black). This is more evident when plotting the mean of the YFP distribution as a function of the distance from the origin of replication. The mean YFP for the LT strain is lower than that expected from the difference in gene copy number (dotted line).
