# Supplementary material for: Gene Regulation by H-NS as a Function of Growth Conditions Depends on Chromosomal Position in Escherichia coli
Source: G3 (Bethesda). 2015 Feb 19;5(4):605–14. doi: 10.1534/g3.114.016139 (PMC4390576; doi:10.1534/g3.114.016139)
Supplement: Supporting Information [file supp_g3.114.016139_FigureS2.pdf]

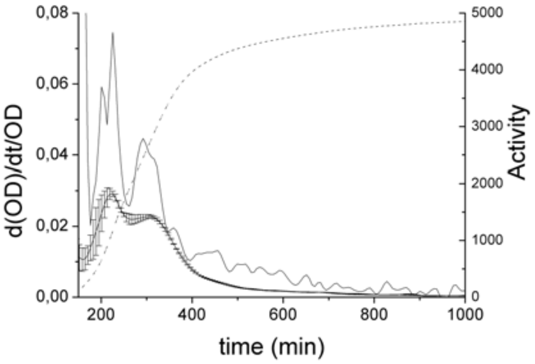

A

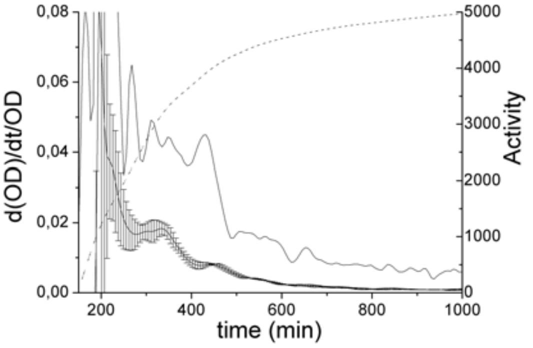

B

Figure S2 The increase in YFP concentration in stationary phase at slow growth (CAA02) is due to the delay in the decrease of promoter activity with respect to the decrease in growth rate. The change in OD is shown by a dashed line, the change in growth rate (d(OD)/dt/OD) in the line with the error bars from the three technical repeats within the experiment and the change in promoter activity (d(YFP)/dt/OD) by a continuous line. (A) Growth rate and promoter activity for cells growing in M9 minimal media supplemented with 0.4% glucose and 0.5% casamino acids (fast growth). The decrease in growth rate at the entry into stationary phase happens at the same time as the decrease in promoter activity, leading to no accumulation of YFP (Fig. 2A main text). (B) Growth rate and promoter activity for cells growing in M9 minimal media supplemented with 0.2% casamino acids (slow growth). The decrease in promoter activity takes place later than the decrease in growth rate, there is therefore an accumulation of YFP at the entry into stationary phase for cells growing in a poorer medium.
