# Supplementary material for: Gene Regulation by H-NS as a Function of Growth Conditions Depends on Chromosomal Position in Escherichia coli
Source: G3 (Bethesda). 2015 Feb 19;5(4):605–14. doi: 10.1534/g3.114.016139 (PMC4390576; doi:10.1534/g3.114.016139)
Supplement: Supporting Information [file supp_g3.114.016139_FigureS3.pdf]

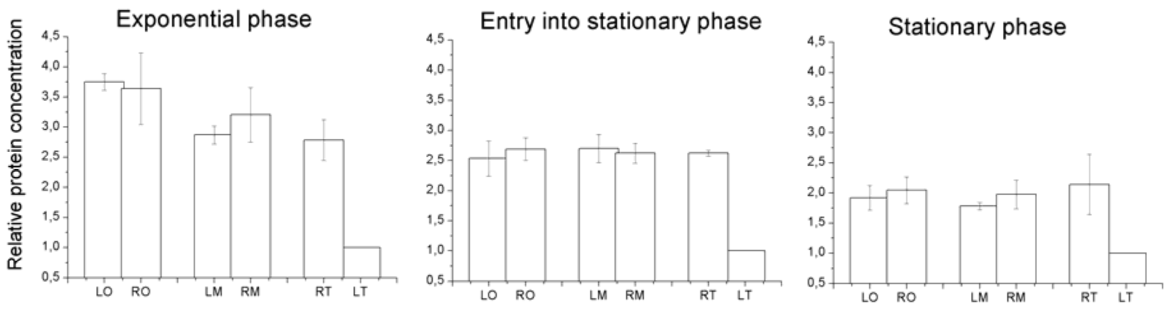

Figure S3 No difference in YFP concentration between RO and LO as a function of growth phase at 37°C compared to 30°C (Fig. 4 in the main text). YFP concentration was normalized by the LT values for strains in exponential, entry into stationary and stationary phase for three independent experiments, the error bars indicate the SEM. Data were taken at the time of maximum growth rate, at the time where the growth rate was half of the maximum and at growth rate equal to zero, respectively. The YFP concentration in the LT strain is always lower than in the other strains. AT 37°C there is no significant difference between LO and RO.
