# Supplementary material for: Gene Regulation by H-NS as a Function of Growth Conditions Depends on Chromosomal Position in Escherichia coli
Source: G3 (Bethesda). 2015 Feb 19;5(4):605–14. doi: 10.1534/g3.114.016139 (PMC4390576; doi:10.1534/g3.114.016139)
Supplement: Supporting Information [file supp_g3.114.016139_FigureS4.pdf]

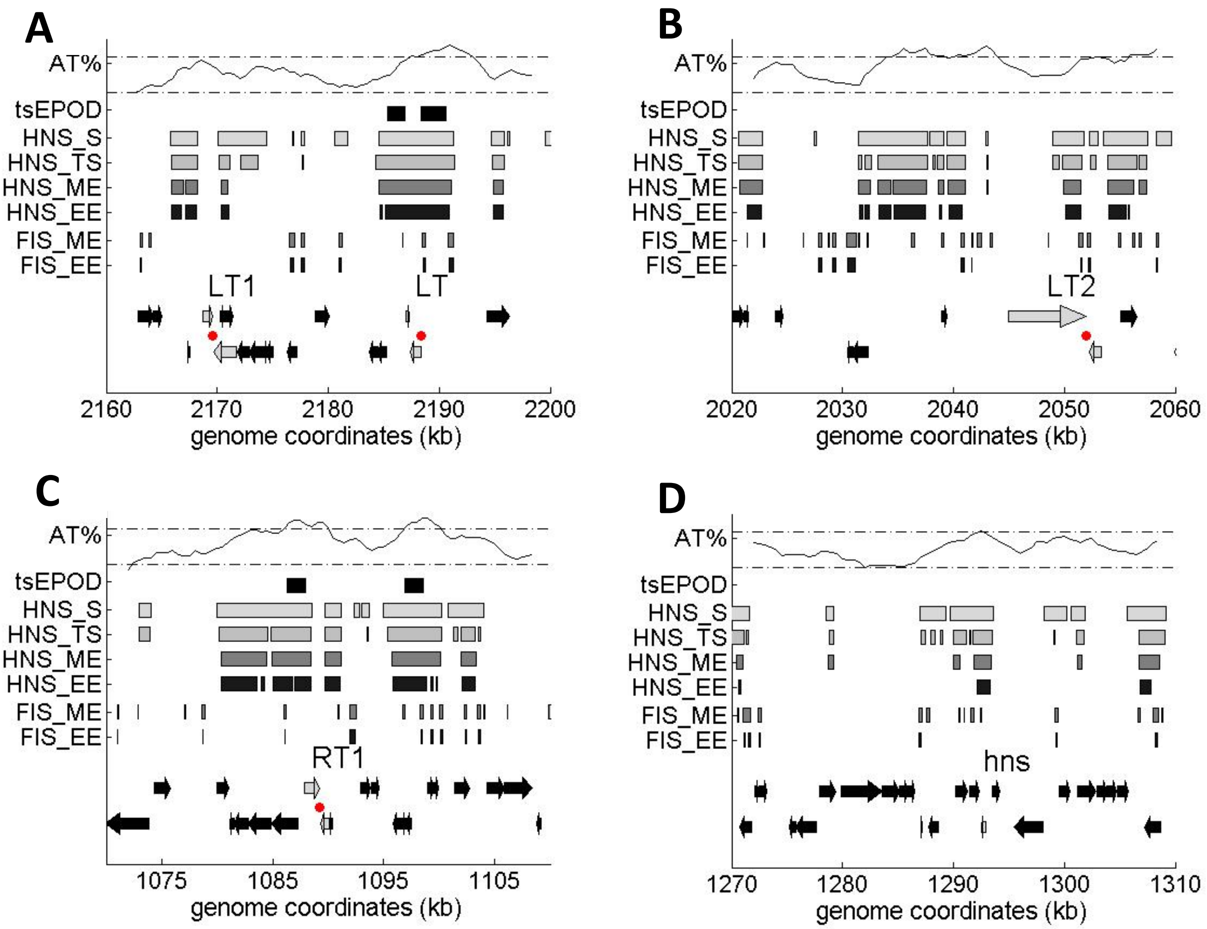

Figure S4 Genomic neighborhood of the additional insertions near LT and RT and of the *hns* gene shows that these sites are found in regions with a higher than average AT content and large H-NS bound regions that increase in size as the cells enter stationary phase. For each plot, from the bottom to the top**:** Genes on the lagging and on the leading strands. In grey are plotted the convergent genes where the insertions have been made, and the *hns* gene itself. FIS binding in early exponential phase (FIS_EE) and in mid-exponential phase (FIS_ME), data from (Kahramanoglou *et al.* 2011). Sites bound by H-NS in early exponential (HNS_EE), mid-exponential (HNS_ME), transition to stationary (HNS_TS) and stationary (HNS_S) phases (Kahramanoglou *et al.* 2011). On the top of these, in LT and in RT1 there are also tsEPOD (Vora *et al.* 2009). AT content is calculated within a 4000 bp sliding window with a shift of 500 bases. In the proximity of all the insertions it is possible to detect a strong occupancy by H-NS as well as a higher AT content of the genomic sequence. For the *hns* gene there is a clear increase in the length of the H-NS bound region while cells are passing from exponential to stationary phase.

Additional references:

Kahramanoglou, C., A. S. N. Seshasayee, A. I. Prieto, D. Ibberson, S. Schmidt *et al.*, 2011 Direct and indirect effects of H-NS and Fis on global gene expression control in Escherichia coli. Nucleic Acids Res. 39: 2073–2091.

Vora, T., A. K. Hottes, and S. Tavazoie, 2009 Protein Occupancy Landscape of a Bacterial Genome. Mol. Cell 35: 247–253.
