# Supplementary material for: Gene Regulation by H-NS as a Function of Growth Conditions Depends on Chromosomal Position in Escherichia coli
Source: G3 (Bethesda). 2015 Feb 19;5(4):605–14. doi: 10.1534/g3.114.016139 (PMC4390576; doi:10.1534/g3.114.016139)
Supplement: Supporting Information [file supp_g3.114.016139_FigureS5.pdf]

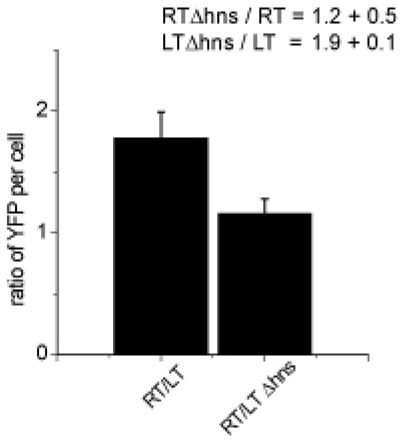

Figure S5 The difference between RT and LT is lost in a Δ*hns* background. The bar graph shows the ratio between the average YFP per cell for the RT and LT insertions in a WT background and Δ*hns* background. Data were obtained by flow cytometry for cells in exponential phase growing in 0.5% casamino acids at 37°C in flasks, shaking. Error bars represent the standard deviation for four independent experiments. The ratio between the fluorescence in RT and LT becomes closer to 1 in the Δ*hns* background with respect to the ratio in the wild type background. This is due to a stronger increase in fluorescence for the LT position in absence of H-NS with respect to the slight increase of fluorescence for RT position in the Δ*hns* background.
