# Supplementary material for: Gene Regulation by H-NS as a Function of Growth Conditions Depends on Chromosomal Position in Escherichia coli
Source: G3 (Bethesda). 2015 Feb 19;5(4):605–14. doi: 10.1534/g3.114.016139 (PMC4390576; doi:10.1534/g3.114.016139)
Supplement: Supporting Information [file supp_g3.114.016139_FigureS6.pdf]

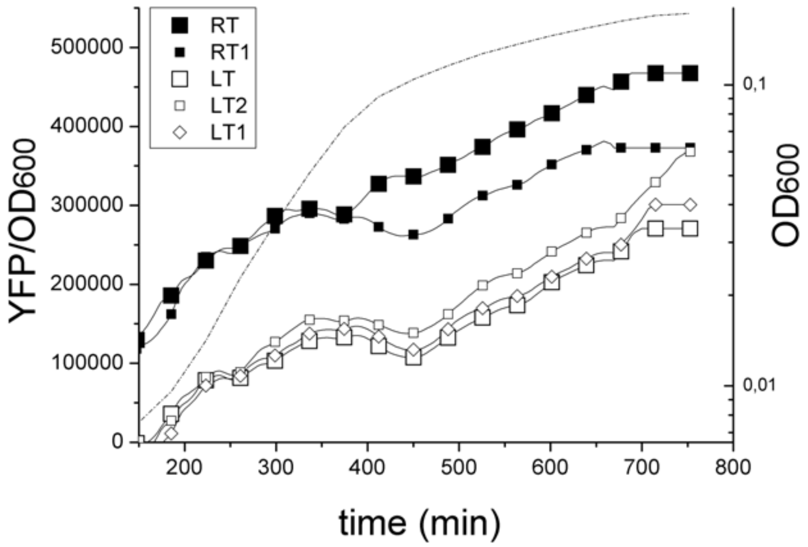

Figure S6 P*hns* promoter activity remains similar for insertions placed up to 135 Kb away from the original sites. Plate reader experiment for strains growing in M9 supplemented with 0.2% casamino acids, at 37°C. The effect of silencing in the LT position extends over several tens of kilobases (LT position= 2185402, LT2=2050038 (Δ135364), LT1=2167635 (Δ17767)), such that the signal from the neighboring strains LT1 and LT2 is similar to the one in LT strain. The YFP concentration in RT1 is similar to the one in RT (RT=1027582, RT1=1093457 (Δ65875)) during exponential phase, while at the entry into stationary phase the expression of RT1 is lower than that of RT.
